# Application of a Novel Hybrid CNN-GNN for Peptide Ion Encoding

**DOI:** 10.1021/acs.jproteome.2c00234

**Published:** 2022-12-19

**Authors:** Kevin McDonnell, Florence Abram, Enda Howley

**Affiliations:** † Department of Information Technology, School of Computer Science, University of Galway, GalwayH91 TK33, Ireland; ‡ Functional Environmental Microbiology, School of Natural Sciences, Ryan Institute, University of Galway, GalwayH91 TK33, Ireland

**Keywords:** *de novo* peptide sequencing, noise, missing peaks, machine learning

## Abstract

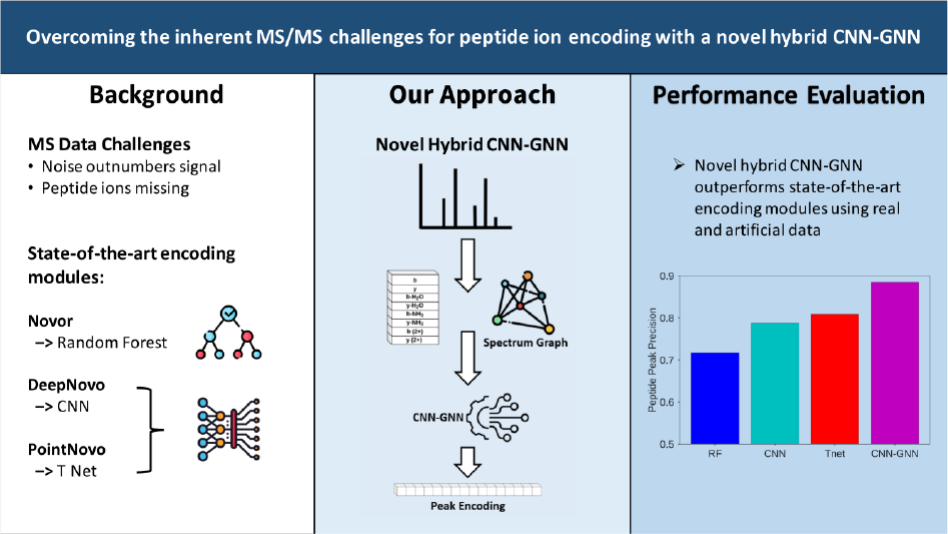

Almost all state-of-the-art *de novo* peptide sequencing algorithms now use machine learning models to encode fragment peaks and hence identify amino acids in mass spectrometry (MS) spectra. Previous work has highlighted how the inherent MS challenges of noise and missing peptide peaks detrimentally affect the performance of these models. In the present research we extracted and evaluated the encoding modules from 3 state-of-the-art *de novo* peptide sequencing algorithms. We also propose a convolutional neural network-graph neural network machine learning model for encoding peptide ions in tandem MS spectra. We compared the proposed encoding module to those used in the state-of-the-art *de novo* peptide sequencing algorithms by assessing their ability to identify b-ions and y-ions in MS spectra. This included a comprehensive evaluation in both real and artificial data across various levels of noise and missing peptide peaks. The proposed model performed best across all data sets using two different metrics (area under the receiver operating characteristic curve (AUC) and average precision). The work also highlighted the effect of including additional features such as intensity rank in these encoding modules as well as issues with using the AUC as a metric. This work is of significance to those designing future *de novo* peptide identification algorithms as it is the first step toward a new approach.

## Introduction

Proteins are large macromolecules which perform essential functions for all life on earth.^1^ They are composed of long chains of amino acids which define their structure and consequently their function. As they are fundamental to all living organisms, the accurate identification of these proteins has wide ranging significance from the detection of cancer^2^ to the optimization of resource recovery from food waste products.^3^ When profiling protein expression (proteomics), the identification process typically involves the enzymatic digestion of the proteins down into smaller sequences of amino acids called peptides. These peptides are then characterized using liquid chromatography tandem mass spectrometry (LC-MS/MS). In this process, peptides of a particular sequence are separated and isolated using liquid chromatography and a mass analyzer. They are then fragmented using collision based methods such as high energy collision dissociation (HCD). For each fragmented peptide sequence, the resulting fragments pass through a second mass analyzer, producing spectra with a unique fragmentation pattern for that sequence. The originating peptides can then be identified from the spectra using a database search.

During the fragmentation process the peptides are generally split between amino acids at the peptide (amide) bonds.^4^ Cleavage at a peptide bond results in b-ions and y-ions. Other ions, such as a-ions, appear when cleavage occurs at other bonds along the amino acid chain. Fragment ions can then suffer neutral losses of both ammonia and water thereby shifting the *m*/*z* of their peaks. Ions can also be doubly charged resulting in *m*/*z* values approximately half that of their singly charged counterparts. As they are made up of subsequences of amino acids, two singly charged peaks of the same ion type from neighboring peptide bonds will be separated in the spectrum at a distance equal to the mass of the amino acid between them. Database search methods work by creating the theoretical peaks for each peptide in the database given the possible fragmentation sites. They then compare these to the peaks in the spectra. A peptide is assigned to a spectrum if its set of theoretical peaks significantly matches the observed array of peaks in that spectrum.

Although widely used, database search methods may only utilize a small fraction of the MS spectra recovered during an experiment.^5,6^ This is partly due to the large protein databases needed to cover the complete set of proteins being investigated.^7^ Larger databases increase the probability of a false positive peptide match therefore increasing the false discovery rate (FDR). To account for this, search algorithms must adopt more stringent criteria for a positive match, thereby excluding many correct but lower scoring matches. For metaproteomics experiments, where there are a large number of possible organisms and therefore even larger databases, the problem is even worse.^8^

*De novo* peptide sequencing is becoming a competitive alternative to these database search methods.^9^ In this strategy, peptides are identified using the spectra alone. This alleviates the need for a database and its associated challenges. An important use of *de novo* peptide sequencing is the identification of neoantigens for cancer immunotherapy,^10^ where peptides specific to a tumor may not be available in a database. The field has benefited tremendously from advancements in machine learning in recent years. Machine learning models have been incorporated into almost all state-of-the-art *de novo* identification algorithms due to their unrivalled pattern recognition capabilities.^11−13^ In this context, machine learning models encode meaningful parts of the spectrum which may help infer the amino acid sequence. The models can learn to differentiate between peptide ions and noise peaks which could be up to 28 times as prevalent.^14^ From this, the algorithms infer the fragmentation sites and thereby the amino acid sequence. However, previous analysis has shown how difficult this inference is due to the aforementioned levels of noise and the even greater challenge of missing ion peaks.^14^ McDonnell et al. found that increasing numbers of fragmentation sites without any representative ion caused an exponential decrease in the accuracy of *de novo* algorithms.

Three such state-of-the-art *de novo* peptide sequencing algorithms that use machine learning are Novor, DeepNovo, and PointNovo.^11−13^ Novor uses a random forest (RF) model to score likely fragmentation sites using ions from neighboring cleavages. DeepNovo and PointNovo use convolutional neural networks (CNNs) to encode these neighboring ions to infer the next amino acid in the sequence. However, as shown in our previous work, these algorithms do not fully encapsulate the fragmentation process.^14^ This is in part due to limitations of their encoding modules. New approaches are needed that can account for this shortcoming.

With many types of machine learning models available to encode peptide ions and each part of a large and complex algorithm, it can be difficult to select the appropriate one when designing *de novo* peptide sequencing algorithms. Therefore, we extract the encoding models from DeepNovo, PointNovo, and Novor and perform a comprehensive evaluation on their ability to identify peptide ions. Also, we propose a novel encoding module, a hybrid CNN-graph neural network (GNN) and compare it to the modules used in these state-of-the-art algorithms. This is the first step toward a new *de novo* sequencing approach. The impact of neighbor independent features on these models is also investigated. Finally we identify issues with the common evaluation metric area under the receiver operating characteristic curve (AUC).

## Background

Peptides are made up of building blocks called amino acids and when they are fragmented using HCD, they generally cleave between these amino acids at the peptide bonds.^15^ Fragmentation at a peptide bond results in b-ions and y-ions, depending on which side of the cleavage the fragment is from. The amino acid sequence of proteins, and hence peptides, are by convention always ordered from the N-terminus to the C-terminus with b-ions relating to the N-terminus fragment and y-ions relating to the C-terminus fragment. Fragmentation sites are also ordered from the N-terminus to the C-terminus. Fragmentation along the peptide chain means that the peaks of fragment ions of the same type appear at intervals from one another, equal to the mass of the constituent amino acids. Through this relationship, the sequence of amino acids can be identified by looking for a sequence of spectrum peaks separated by amino acid masses. Identification of these fragment ions is therefore essential to the *de novo* prediction of peptides.

As they cannot rely on a database to know which spectrum peaks correspond to fragment ions, *de novo* algorithms look at features of each peak as well as their relationship to other peaks to distinguish likely candidates. The amino acid sequence is then built up by moving from one peptide peak (fragment ion) to the next. The step size between peaks indicates the amino acid in the sequence.

Before fragmentation the ionized peptides are separated by their mass in the first mass analyzer. The ions that are selected for fragmentation are called the “parent ions” as they are broken down into fragment ions. While the peptide sequence is not known, its mass can be inferred from the parent ion. The mass of complementary b-ions and y-ions that came from the same fragment site can then be identified using the following formulas:
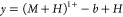
1
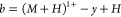
2where *y* is the mass of the y-ion, *b* is the mass of the b-ion, *M* is the mass of the peptide, and *H* is the mass of a hydrogen atom.

These can be generalized to the formula:
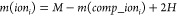
3where *m*(*ion*_*i*_) is the mass of an ion from the *i*^*th*^ fragmentation site and *m*(*comp*_*ion*_*i*_) is the mass of the complementary ion from the *i*^*th*^ fragmentation site.

Ions of the same type from neighboring fragmentation sites can be identified using the following formula:
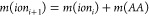
4where *m*(*AA*) is the mass of an amino acid.

Combining eqs 3 and 4 to look for complementary ions from a neighboring fragmentation site, we get the following:
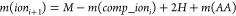
5

Some ions lose a neutral molecule of H_2_O or NH_3_. While the charge is maintained the resulting *m*/*z* is shifted by the corresponding mass of the lost molecule. This can then be incorporated into the above equations by subtracting this mass from the ion in question. The following shows the case for the loss of H_2_O:
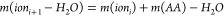
6

Ions can also be doubly charged (*ion*^2+^). To convert a doubly charged peak to its singly charged form one only needs to double its *m*/*z* value and subtract the mass of the extra hydrogen nucleus (proton):
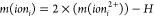
7

Ions of other types can occur if fragmentation occurs at bonds other than the amide bond. While these are less likely to occur under HCD conditions, they can easily be incorporated into the search space. N-terminus ions can be calculated with respect to the b-ion by taking into account the relevant atomic differences. For instance a-ions can be identified by subtracting the mass of CO (28 Da) from the corresponding b-ion. In a similar way, the other C-terminus ions can be identified with respect to the corresponding y-ion.

The *de novo* peptide identification problem can be solved by creating a graph with every peak as a node.^16^ The above equations are then used to create connections between nodes from potential neighboring fragmentation sites. Passing through the graph from zero to the mass of the parent ion, a peptide sequence will emerge from the amino acid connections used to create the path.

*De novo* peptide sequencing is not without its difficulties, however. Peaks not attributable to the peptide (noise) account for the vast majority of peaks in tandem MS spectra.^14^ Furthermore, peaks corresponding to all possible fragment ions from a peptide may not appear in the spectrum. This is particularly problematic when no ion from a fragmentation site is present leading to ambiguity in the order or identification of the amino acids. The absence of fragment ions can be partly attributed to the fact that the cleavage of some peptide bonds may be less energy favorable than others.^17^

The machine learning modules of *de novo* peptide sequencing algorithms create encodings which are used to indicate the likelihood of a fragmentation site or particular amino acid at that position. The modules encode the intensity of the peaks and those near them defined by eqs 4 and 5 as well as other features in the spectrum.

Tiwary et al.^18^ showed that there is a significant relationship between the ion intensity and the complete amino acid sequence, not just the neighboring cleavages. However, in many *de novo* algorithms, only peaks from possible neighboring peptide bond cleavages are considered as the number of possible locations grows exponentially for cleavages farther away. These long-range interactions should be considered in future *de novo* peptide identification algorithms. GNNs are a great way to encapsulate the long chain-like structure of the peptide without an exponential increase in complexity, but so far no algorithm has utilized them in the context of *de novo* peptide identification.^14^

Novor is a *de novo* peptide sequencing algorithm that uses an RF machine learning model.^11^ The RF model encodes related peaks to score the likelihood of a fragmentation site. It also uses other features of peaks such as their intensity rank to influence its decision. Using the above equations, the mass difference between fragmentation sites can be used to identify amino acids. Novor then uses dynamic programming to find the highest scoring combination of fragmentation sites that fulfill the peptide mass.

DeepNovo is a more recent *de novo* algorithm using both CNNs and a long short-term memory network (LSTM) to encode peptide ion peaks as it steps through the spectrum.^12^ Starting at one end of the peptide, the algorithm looks to identify each amino acid in the sequence one-by-one. Given its current position, the CNN encodes the sections of the spectra where the next possible peptide peaks may occur (eqs 4 and 5). The output of the CNN is then passed to an output layer or LSTM to identify next most likely amino acid. Dynamic programming is also used to limit the number of possible amino acids that can be predicted. Following this, the current position is updated to either that of the b_*n*_-ion or y_*n*_-ion given the *n* amino acids already predicted and the direction of prediction. While DeepNovo’s LSTM can use information from previously traversed peaks, its encoding module does not have any way of looking more than 1 amino acid ahead. Also, as stated in the original paper, the LSTM only uses the previous two amino acids to influence its decision. This is done by resetting the LSTM using the output of a second CNN which transforms the spectrum into a vector encoding. At each prediction step, this vector is then used to initialize the LSTM before the previous two amino acid predictions are encoded. This was found by the authors to reduce overfitting.^12^ Although DeepNovo does use a beam search to explore a greater number of possible sequences, each is generated using this limited information.

An updated version of DeepNovo has been released called PointNovo.^13^ The methodology of this approach is very similar to its predecessor. However, the CNN used to encode the spectrum windows is replaced by a T Net which uses absolute peak differences and not spectrum sections. The T Net is essentially a 1-dimensional CNN with a kernel size of 1. The T Net encodes both the difference between each peak and the theoretical position of neighboring fragment ions as well as the intensity of each peak. Again, this encoding can be either passed to an LSTM or an output layer to predict the next amino acid in the sequence. Unlike its predecessor, the LSTM used by PointNovo encodes all previous amino acid predictions. Also, as it uses the difference values and not discretized windows, PointNovo is more robust to different resolution mass spectrometers.^13^

Our previous work showed limitations in the approaches of modern algorithms.^14^ The peptide accuracy of the models was found to decrease exponentially with increasing numbers of missing fragmentation cleavages. Also, DeepNovo showed a much steeper decline in amino acid recall than Novor as the number of missing fragmentation cleavages increased. This led to Novor performing better for spectra with more than 4 missing cleavages. This previous work highlighted the need to explore different methodologies to address the problems of *de novo* peptide sequencing while also showing a potential avenue of exploration.

The work described here constitutes the first step in the exploration of such new methodologies with the use of GNNs for peptide ion encoding. GNNs can capture the graph like structure of the peptide fragmentation process as well as having the pattern recognition capabilities of neural networks. Therefore, their architecture can encode more spectrum information than CNNs alone. However, the following evaluation does not involve the integration of GNNs into the aforementioned algorithms. The GNN module proposed updates all nodes simultaneously. This is in contrast to the step-like architecture of DeepNovo and PointNovo and so it does not easily fit into their algorithm. Furthermore, the code of Novor is not open source and so we cannot integrate our model into their architecture either. Nevertheless we wish to benchmark this approach against other encoding modules used in this space. As such we compare a novel CNN-GNN approach with the encoding modules of the three state-of-the-art *de novo* peptide sequencing algorithms on their ability to identify peptide ions.

This means that the encoding modules employed by DeepNovo and PointNovo will not be doing exactly what they were designed to do. While it may seem likely that peptide ion identification and amino acid identification are related, this has not been explicitly shown in this research. Nonetheless, *de novo* encoding modules should be designed to learn features of spectra that link observed fragmentation patterns to the corresponding peptides. As outlined earlier, identification of the chain of backbone ions can elucidate the peptide. Therefore, effective encoding modules should be able to identify ions from this chain. While a step-by-step approach is currently the state of the art, perhaps a more complete spectrum encoding is required. This research proposes integrating the long-range relationships between backbone ion peaks into the encoding process through the use of GNNs. The aim is to highlight the potential of GNNs in the context of peptide ion identification and hence *de novo* peptide identification.

## Methods

### Benchmark Data Sets

Real HCD tandem mass spectra, collated by Tran et al.,^12^ were used in this evaluation. HCD data provides greater resolution and mass accuracy than other fragmentation methods.^19^ The data are available to download at ftp://massive.ucsd.edu/MSV000081382/. The data are made up of tandem mass spectra from 9 different organisms from 9 different research groups.^20−28^ The spectra were labeled by Tran et al. with peptides using a database search with a 1% FDR threshold against the UniProt database.^29^ These peptides are assumed to be correct, with their fragment ions serving as ground truth labels in this research (see next section). Table 1 lists a summary of the data sets. More details including the precursor and fragment tolerances used are available in the original paper.^12^

**Table 1:** Summary of Real Data Sets Used^a^

Data Set	Organism	Mean FPP	Mean NR
Yeast	*Saccharomyces cerevisiae*	0.37	6.3
Human	*Homo sapiens*	0.24	5.0
Mouse	*Mus musculus*	0.21	4.1
Bacillus	*Bacillus subtilis*	0.35	6.4
ClamBacteria	*Candidatus Thiodiazotropha endoloripes*	0.22	3.7
Honeybee	*Apis mellifera*	0.35	7.4
Ricebean	*Vigna mungo*	0.28	6.0
Tomato	*Solanum lycopersicum*	0.30	4.4
M. mazei	*Methanosarcina mazei*	0.31	6.5
All Data		0.29	5.6

a FPP is the fraction of peptide peaks present in the spectra. NR is the ratio of noise peaks to peptide peaks.

Data partitioning into training, validation, and test sets was also carried out Tran et al. This was done by having separate partitions for each organism. The training and validation data for each organism type were made up of spectra from the other 8 data sets. Then, testing was then done on spectra from the organism itself, essentially performing a leave-one-out cross-validation. Each test set was made up of approximately 10 000 spectra from a single organism. Nine models were trained for each encoder type tested in this evaluation, one for each organism type.

### Peak Classification

Theoretical peaks were created for each peptide using the Pyteomics module.^30^ These were then compared to the peaks in the corresponding spectra. Peaks were labeled as peptide peaks if they matched the theoretical peaks within a tolerance of 0.05 Da.

The ions considered were b-ions, y-ions, b-H_2_O ions, y-H_2_O ions, b-NH_3_ ions, y-NH_3_ ions, b(2+) ions, and y(2+) ions. Peaks that could not be assigned to one of these ion types were labeled as noise. If multiple peaks fell within the tolerance only the peak with the smallest error was assigned an ion. The number of possible/theoretical peaks, given these ion types, is known for each peptide. Each spectrum was then classified by the fraction of theoretical peptide peaks present (FPP) by matching them to the observed spectrum peaks. The number of peaks that remained unmatched (noise) were compared to the number of identified peaks. The relative proportion of these for each individual spectrum we define as the noise ratio (NR).

### Artificial Data Sets

Additionally, models were also evaluated on artificial data created using the Prosit pipeline.^31^ Prosit creates artificial spectra with accurate representations of both the mass and intensities of peptide peaks. As it is a well studied model organism, *Saccharomyces cerevisiae* was selected as the basis for the artificial data. The yeast proteome (UP000002311) was downloaded from Uniprot on 02/07/2021.^29^ Protein sequences were artificially digested using the Pyteomics parser.^30^ Artificial spectra were created for each unique peptide (188 694) using the Prosit pipeline. To create a manageable data set size and match the real data test set sizes, a random sample of 10 000 spectra were selected as the artificial data set.

The artificial data set has a mean FPP equal to 0.36. The FPP of the artificial data set is not 1.0 as Prosit does not predict peaks that would be very unlikely to appear in real spectra. However, while the value closely matches that of the real yeast data set (0.37), the distribution of ion types and numbers matched are quite different (data not shown). The models were then evaluated on the artificial data with adjusted levels of noise. The Prosit data set was duplicated 5 times. As it was duplicated the peaks present were the same for each data set with only the level of noise changing. Then to span the range of values in real data, additional noise was added at ratios to the peptide peaks of 0, 1, 5, 10, and 15.

The *m*/*z* values of the artificial noise peaks were randomly sampled from a uniform distribution between zero and the mass of the peptide attributed to the spectrum. The intensity values of the artificial noise peaks were randomly sampled from a distribution approximating the noise intensity values in the real yeast data set. This approximation is a log-normal distribution. The mean and standard deviation of the natural log of this distribution is −4.4 and +1.5, respectively.

### Ion Identification

Machine learning modules in Novor, DeepNovo, and PointNovo all encode fragment ions in reference to the positions of possible b-ions and y-ions. These encodings then inform either the likelihood that the current peak is from a fragmentation site or the prediction of the next amino acid in the sequence. In the case of DeepNovo and PointNovo, their algorithms move to the position of the next b-ion and y-ion in the spectrum depending on the latest amino acid predicted and the direction of prediction. In this research, models are evaluated on their ability to identify these b-ions and y-ions using the features listed in the next section. Each module was used to encode the relevant spectrum sections to vector of length 512, the same size used as DeepNovo. This was then followed by a single output node to give a score to each peak that it is one of the two ions. Models that could encode more information should learn to better distinguish these ions from the other peaks.

### Model Features

Features used by the state-of-the-art models were extracted from the spectra as follows. Assuming each peak corresponds to a b-ion or y-ion, sections of the spectrum surrounding the following locations were identified; the location of all possible peptide ion peaks from cleavages in front of the current ion (eqs 4 and 5), the location of all possible peptide ion peaks from cleavages behind the current ion (eqs 4 and 5 with changed signs), and the location of the possible complementary ion peak in the spectrum (eq 3). Neutral loses of H_2_O and NH_3_ as well as double protonation were considered for each ion type. To align with the precision of the algorithms, 0.1 Da windows surrounding the exact positions (0.05 Da each side) were extracted and peaks were placed into the relevant bins, each of size 0.01 Da. The forward and backward ion features have the shape (#AA, #ion types, window size) where #AA is the number of possible amino acids and their modifications (26), #ion types is the number of ion types (8; b-ions, y-ions, b-H_2_O ions, y-H_2_O ions, b-NH_3_ ions, y-NH_3_ ions, b(2+) ions, and y(2+) ions), and window size is 10 (0.1/0.01). The features used by the T Net are slightly different in that instead of discretized windows the absolute difference is used. This is to correspond with the features and module used in the original paper.^13^

Alongside neighboring peaks, Novor also uses expert selected features to enhance model accuracy.^11^ These include the peak intensity rank, peak intensity half rank, local intensity rank and local intensity half rank. The addition of these features was included in the models denoted by “+F”. For more information on these features see the original paper.^11^

### Random Forest Model

An RF model was created to emulate the scoring function of the Novor algorithm.^11^ The RF model is used by Novor to measure the probability each peak is from a real fragmentation site. The RF evaluated in this research is an approximation of the model used by Novor as the source code is not released. A further description of the Novor algorithm can be found in the original paper.^11^ The RF model was trained with the above features (see previous section) as input and a single variable output. It was created using the scikit-learn^32^ library. The number of trees in the model was set to 1000. There was no maximum depth for the trees. As Novor uses the additional features in its algorithm, the RF model was only evaluated with these features included (RF+F).

### CNN Model

Similarly a CNN model was created to emulate the encoding module of DeepNovo’s algorithm.^12^ The original code can be found at https://github.com/nh2tran/DeepNovo. A complete description of the DeepNovo algorithm can be found in the original paper.^12^Table 2 lists a summary of the CNN module. Just like DeepNovo, the first convolution layer in the module has a 1 × 3 filter and a stride of 1 in both directions. The second layer has a 1 × 2 filter again with a stride of 1 in both directions. the Max_pool layer has a filter size of 1 × 3 and strides of (1,2). Finally the layers are flattened and passed to two consecutive dense layers of size 512. It should be noted that in addition to the amino acids, DeepNovo encodes Start, End, and Pad tokens. These are set to zero and ignored.

**Table 2:** Structure of Each CNN Module as used by DeepNovo

Layer	Output Shape
Input Window	(None,26,8,10)
Transpose	(None,8,10,26)
Conv2D_1	(None,8,10,64)
Conv2D_2	(None,8,10,64)
Max_pool	(None,8,5,64)
Dense	(None,512)
Dense	(None,512)

Three of these CNN modules were used in the complete model, one for each spectrum window (forward, backward, and complementary). The output of the three CNNs are concatenated and passed to a 512 dense layer before a single node output layer. The model was created with (CNN+F) and without (CNN) the additional Novor features. The CNN models were trained using focal loss^33^ as recommended by Tran et al.,^34^ and the Adam optimization algorithm.

### T Net Model

A T Net module was created to emulate the encoding module of PointNovo.^13^ Code for the original implementation can be found at https://github.com/volpato30/DeepNovoV2 with further information in the original paper.^13^ While PointNovo essentially uses the same features as DeepNovo it formats them differently. Instead of spectrum windows the T Net model uses the differences from the theoretical values of each possible amino acid. The T net encodes the differences and intensities of all the possible amino acids with each peak in the spectrum. A sequence of three one-dimensional CNNs converts the input into a 64-dimensional vector followed by two consecutive dense layers of size 512.

Three T Net modules were combined with one each for forward, backward, and complementary ions. These were then combined and condensed using a further 512 dense layer as with the CNN above.

PointNovo uses 12 ions which includes a-ions, a-H_2_O ions, a-NH_3_ ions, and a(2+) ions, in addition to the 8 ions listed above. Therefore, the T Net was included in both 8 (Tnet8+F) and 12 (Tnet12+F) ion versions. Both versions include the additional features denoted by “+F”. All T Net models were trained using focal loss and the Adam optimization algorithm.

### CNN-GNN Hybrid Model

The proposed encoding module uses a GNN to capture the long-range interactions of tandem peptide spectrum graphs. In a GNN with a graph , the embedding for each node , is defined as , where *k* is the number of message passing layers i.e. update steps. The embedding  is updated by aggregating the embedding of *u*’s neighbors . The proposed GNN uses a mean aggregation of the neighbor embeddings for each node, given by the following equation:
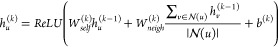
8where *W* is a weight matrix and *b* is bias vector. The architecture was inspired by the encoder module of the Graph2Seq model.^35^

The CNNs described above are used to create the node embeddings. As before, at each peak, spectrum windows which encompass the possible neighboring amino acids are passed to the three CNNs. Again, the output of the three CNNs is concatenated before passing through a 512 dense layer. This provides the initial node embeddings . A graph is then created with a node for each peak and edges between nodes where eqs 4 and 5 are satisfied. An aggregate path length of 4 is used as a compromise between complexity and performance. At each node the embeddings of its neighbor and itself are combined and then updated using the above formula (eq 8), specifying the new embeddings for each node. The process is repeated 4 times resulting in a 512 vector encoding for each node. These GNN encodings are then passed to a single node output layer to provide the peak score. The CNN node embedding and aggregation steps of the GNN are all trained together. For simplicity, the CNN-GNN hybrid will be referred to as just GNN for the rest of the paper. The model was created both with (GNN+F) and without (GNN) the additional Novor features. Like the other neural network models, all GNN models were trained using focal loss and the Adam optimization algorithm.

### Model Evaluation

Both the area under the precision-recall curves (AUPR) and the AUC were considered as metrics in this research. AUPR summarizes the precision-recall curve into a single number. Both recall (eq 9) and precision (eq 11) are independent of the number of true negatives (TNs). This makes AUPR a more informative metric when the negative class (noise) vastly outnumber the positive class (peptide ions).^36^ AUPR is difficult to calculate, however, as a linear interpolation between points in PR space leads to an overestimation of performance.^36^ Therefore, we use an approximation of the AUPR, namely the average precision over all score thresholds.^37^ Average precision was calculated using the metrics module for Scikit-learn.^32^

AUC was also used in the evaluation. The receiver operating characteristic (ROC) is the plot of the true positive rate (TPR; eq 9) against the false positive rate (FPR; eq 10). AUC, the area under this curve, is a popular metric for binary classification tasks such as those described in this research as it also captures the performance of a model in a single statistic.^38^ AUC can be interpreted as the probability that a model will score a randomly selected positive example higher than a randomly selected negative example.
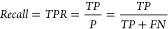
9
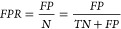
10
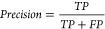
11

### Hardware Specifications

All models were trained on a 16 GB linux machine with an Intel(R) Core(TM) i7-9750H CPU @ 2.60 GHz and Nvidia GeForce GTX 1650 4 GB GPU. Random forest models were created using the Scikit-learn 0.24.1 module while deep learning models were created using Tensorflow 1.13.1.

### Code Availability

The artificial data and deep learning models used in this research are available at https://github.com/KevinMcDonnell6/MSencoding.

## Results and Discussion

### Performance on Benchmark Data sets

All models were evaluated on 9 real tandem MS data sets from 9 different organisms. Table 3 shows the average precision of the models on all 9 data sets. The GNNs with and without the additional features were the top two performing models in all data sets. The GNN+F was found to perform best in 8 of the 9 data sets with the standard GNN performing slightly better on the Tomato data. The RF+F was the worst performing model in all but one of the 9 data sets with the CNN marginally worse on the Human data set.

**Table 3:** Average Precision Values for Each Model on All 9 Real Data Sets

Data Set	RF+F	CNN	CNN+F	Tnet8+F	Tnet12+F	GNN	GNN+F
Yeast	0.7170	0.7368	0.7873	0.8110	0.8092	0.8612	0.8853
Human	0.7235	0.7234	0.7926	0.8048	0.8081	0.8472	0.8679
Mouse	0.6848	0.7287	0.7648	0.7942	0.8041	0.8466	0.8641
Bacillus	0.6572	0.6878	0.7558	0.7619	0.7759	0.8333	0.8567
ClamBacteria	0.7049	0.7146	0.7864	0.7872	0.8070	0.8290	0.8548
Honeybee	0.6375	0.6563	0.7248	0.7418	0.7660	0.8004	0.8299
Ricebean	0.6636	0.6754	0.7435	0.7408	0.7534	0.8249	0.8516
Tomato	0.7403	0.7666	0.8246	0.8365	0.8428	0.9017	0.9002
M. mazei	0.6613	0.6901	0.7529	0.7693	0.7813	0.8490	0.8586

The results demonstrate the strength of the graph approach in assisting peptide peak identification. The graph can encapsulate more information as long-range interactions are passed through and encoded by the model. The GNN maintained a significantly higher average precision than the other models despite the variation between the data sets of organism type, FPP, and NR (Table 1). This indicates a robustness in the GNN architecture regardless of the data characteristics it is faced with. However, as of yet they are unused in the context of *de novo* peptide identification. The advantage of GNNs is that they can capture the inherent graph-like nature of peptide fragmentation patterns. Consequently, these results suggest their utility in *de novo* peptide sequencing should be explored further.

The results also show the utility of Novor’s additional features. These expert features devised by Novor include intensity rank, intensity half rank, local intensity rank, and intensity half rank. The CNN+F performed substantially better than the standard CNN for all data sets. The superiority of models that utilize these features highlight that there is still a place for expert knowledge in the field of *de novo* peptide sequencing. Machine learning is often seen as being able to provide a solution to all problems. However, care must be taken when designing architectures and/or features that best fit the problem at hand. In this context area experts can still play an integral part in machine learning algorithm design. Also, while these features are useful in distinguishing peptide peaks from noise, it is unclear whether or not they can assist in amino acid prediction as well. The features were designed and utilized by Novor to identify fragmentation sites. Future algorithms similar to DeepNovo may benefit from expert features designed for their step-by-step amino acid prediction approach.

### The Effect of Missing Peaks

To further investigate the above results, the performance of the models was compared for spectra with varying amounts of noise and peptide peaks present. In our previous work these were found to be the main challenges when identifying peptides *de novo*.^14^

To do so, all 9 real data sets were merged and each spectrum was assigned a NR and FPP based on the amount of ion peaks identified (see section Peak Classification). The FPP values spanned the range from 0 to 0.8 with spectra grouped into bins encompassing each 0.1 span. The NR values ranged from 0 to 30 with spectra grouped into the designated bin for each 1.0 increase. The final bin encompasses the NR range from 14 to 30 as very few spectra matched this range.

**Figure 1:**
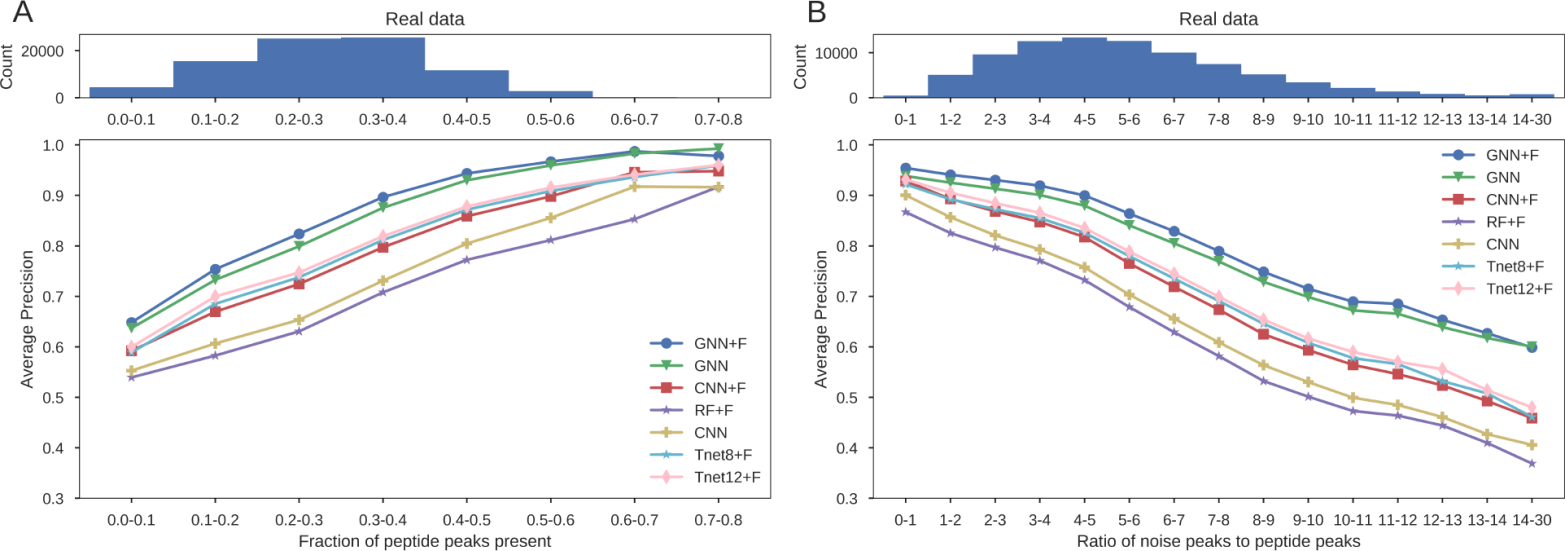
Performance of models with respect to the fraction of peptide peaks present and noise ratio. Average precision is shown for spectra matching the different grading of both features.

Figure 1A shows how the performance of the models changes with respect to the fraction of possible peptide peaks present. Spectra were grouped into bins corresponding to fraction of peaks present. Average precision was then calculated for each bin as shown. When many peptide peaks are missing, the performance of the models is the worst. As the fraction of peptide peaks present increases, so does the average precision.

Consistent with the overall results from the 9 data sets, the GNNs were the best performing models across all of the data. Even when many peaks were missing (FPP < 0.1) the graph approach performed the best. While the complete chain of ions may not be available, partial chains help inform ion identification. This aligns with our previous findings where despite many fragmentation sites not being represented in the data, Novor was able to make use of the partial sequences of sites that were.^14^*De novo* algorithms should be designed so that they can utilize partial sequences and not rely on complete ion chains to be present as these data account for only a fraction of the total.

The advantage of the additional expert features is again evident with those models utilizing them performing better than their counterparts for almost all of the data. The CNN+F performs better than the CNN for all data across the range of peptide peak prevalence. The GNN without these features surpasses the GNN+F only when very few peaks are missing. While there is very little data in this range the performance of these models converges as the fraction of peaks present increases. The advantage of the features becomes minimal when the graph is almost complete and information can pass between peaks instead of relying on the independent features. Conversely, the CNN+F maintains its advantage over the CNN in the same range. These models are not able to encode the long-range interactions and hence the additional features have a greater impact.

### The Effect of Noise

The models were also evaluated with respect to the ratio of noise to peptide peaks in the spectra (Figure 1B). Noise is defined as any peak that could not be attributed to a b-ion or y-ion in the database assigned peptide, either singly or doubly charged, or singly charged with a neutral loss of water or ammonia. Spectra were again binned, this time corresponding to their noise ratio. Average precision was calculated for each bin as shown.

The GNN+F was found to be the best performing model for almost all noise ratios with the GNN without additional features the next best. The increase in precision of the GNNs over the other models was the greatest when noise ratios were high.

When many noise peaks are present, some of them may appear by chance at mass values equal to an amino acid away from a peptide peak. It is very difficult for the CNN, T Net and RF to distinguish these from actual peptide peaks. The GNNs have the advantage of being able to encapsulate long-range relationships between peaks in the spectrum and so are better able to distinguish the real peptide peaks from noise. Real peptide peaks are likely to appear as part of a chain of peaks, which is extremely rare for noise.

The effect of noise was also investigated using artificial data (Table 4). Artificial data provide a way to evaluate the models on the same data while changing only the number of nonpeptide peaks. The yeast proteome downloaded from Uniprot^29^ was used to create a list of peptides. A data set of the corresponding spectra was created using the Prosit pipeline.^31^ This data set was duplicated and then the noise ratio was artificially set to the specified levels for each duplicate. The noise ratio of each spectra was artificially assigned thereby controlling for the correlations between variables observed in real data (Figure S1). The models prepared for the real yeast data, which were therefore not trained on any yeast spectra or peptides, were used in the evaluation.

**Table 4:** Average Precision Values for All Artificial Data Sets^a^

Data Set	RF+F	CNN	CNN+F	Tnet8+F	Tnet12+F	GNN	GNN+F
FPP0.36 NR0	0.9865	0.9972	0.9972	0.9948	0.9974	0.9984	0.9981
FPP0.36 NR1	0.9805	0.9943	0.9947	0.9964	0.9966	0.9964	0.9977
FPP0.36 NR5	0.9341	0.9759	0.9727	0.9875	0.9791	0.9866	0.9915
FPP0.36 NR10	0.8749	0.9403	0.9268	0.9648	0.8091	0.9702	0.9791
FPP0.36 NR15	0.8191	0.8937	0.8774	0.9223	0.6163	0.9491	0.9611

a FPP stands for fraction of peptide peaks present and NR stands for noise ratio.

Table 4 shows the average precision values for the yeast models on the 5 artificial data sets. Like the real data, the GNNs were the top two performing models for each data set. The GNN+F was the best model for all data sets except when there was no additional noise and the GNN performed marginally better.

### CNN-GNN Hyperparameter Comparison

The GNN+F model was also trained and tested with different hyperparameters, such as the number of message passing layers, the direction of neighboring nodes to aggregate, and the aggregation function.

Table 5 lists how average precision increases with increasing numbers of message passing layers but with decreasing magnitude. The GNN model is designed such that setting the model to have 0 message passing layers is equivalent to the CNN model. As shown in Table 5 increasing the number of layers from 0 to 2 gives an initially large increase in average precision of 11%. Further increases from 2 to 4 and 4 to 6 give more modest improvements of 1.2% and 0.88% respectively.

**Table 5:** Average Precision Values for Different GNN+F Models on the Yeast Data Set^a^

#Layers	Aggregation Fn	Direction	Average Precision
0	Mean	Fw and Bw	0.7873
2	Mean	Fw and Bw	0.8748
4	Mean	Fw and Bw	0.8853
6	Mean	Fw and Bw	0.8931
4	Sum	Fw and Bw	0.8759
4	Mean	Fw	0.8766
4	Mean	Bw	0.8740

a The number of aggregation layers is denoted by #Layers, the aggregation function is specified under Aggregation Fn and the directions information could flow is highlighted under Direction.

Table 5 also shows how mean aggregation was found to give better average precision than sum aggregation when there are 4 message passing layers. Mean aggregation is more stable to differing node degrees as it normalizes the inputs maintaining the same scaling. Models also tended to converge to their optimum quicker using mean aggregation (data not shown).

Models with 4 layers were also also trained using only the forward or backward connections in the graph. In this context, forward connections refer to nodes from the apparent succeeding cleavage (eqs 4 and 5), with backward connections from the preceding cleavage. The results show that both 4 layer unidirectional models exhibited similar average precision to each other but lower than the 4-layer model using both directions. The average precision of the 4-layer single direction models is very similar to that of the model utilizing both directions over only 2 layers. For any given peak, each of these 3 models are using neighbor encodings that span a distance of 4 hops. Although each model would ultimately be using different neighbors the results remain consistent.

### Problems with AUC

During the evaluation of the models, AUC was also used as a metric to compare their performance across the different data sets. However, this analysis resulted in some unusual findings.

**Figure 2:**
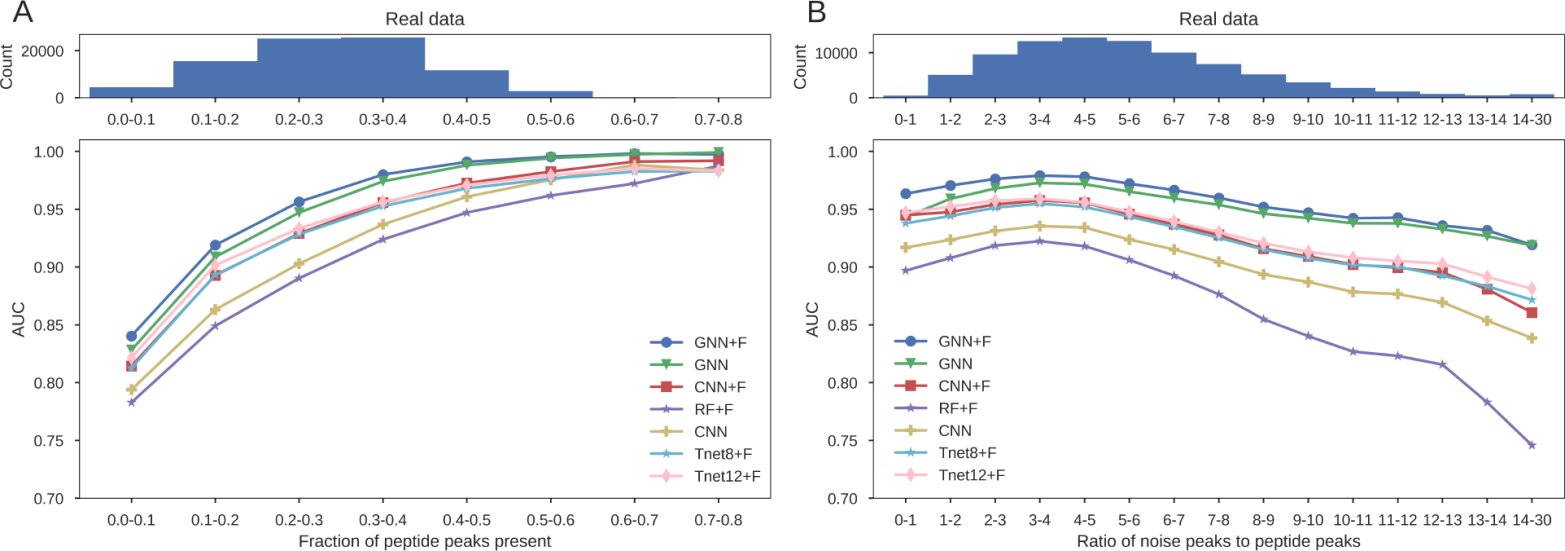
Performance of models with respect to the fraction of peptide peaks present and noise ratio. AUC is shown for spectra matching the different grading of both features.

Figure 2 shows how the AUC changes for each model on the real data when binned by the fraction of peptide peaks present and the noise ratio. There is an initial increase in AUC as the noise ratio increases from 0 to 5 for all models (Figure 2B). This is in contrast to previous findings which showed increasing noise levels resulted in worse performance.^9,14^

Upon further investigation it was found that this portion of the data had a much lower than average fraction of peptide peaks present, which may contribute to the lower than expected performance overall (Figure S1A). As shown in Figure 1A, lower levels of peptide peaks present correlate to lower overall performance. However, if this was the case it did not affect the average precision (Figure 1B).

To account for these correlations the AUC was calculated for each model on the artificial data where only the noise level was changed (Table 6). Again an initial increase in the AUC was observed for each model as noise was added to the data.

**Table 6:** AUC Values for All Artificial Data Sets^a^

Data Set	RF+F	CNN	CNN+F	Tnet8+F	Tnet12+F	GNN	GNN+F
FPP0.36 NR0	0.9609	0.9918	0.9916	0.9841	0.9920	0.9954	0.9946
FPP0.36 NR1	0.9861	0.9960	0.9961	0.9973	0.9974	0.9969	0.9984
FPP0.36 NR5	0.9813	0.9941	0.9924	0.9964	0.9951	0.9948	0.9976
FPP0.36 NR10	0.9736	0.9896	0.9839	0.9917	0.9863	0.9918	0.9957
FPP0.36 NR15	0.9670	0.9836	0.9758	0.9875	0.9762	0.9883	0.9932

a FPP stands for fraction of peptide peaks present and NR stands for noise ratio.

Investigation into the definition of AUC provided some insights as to why this is the case. The additional noise introduces many easy to classify negative examples. This increases the size of the negative class (N) while having little effect on the number of false positives (FPs) (see eq 10). This lowers the false positive rate thereby inflating the AUC. Conversely, neither the precision or recall are proportional to the size of the negative class (eqs 9 and 11). This is why average precision does not show similar trends. Further discussion can be found in the Supporting Information.

### Time Evaluation

Increased performance may come at a cost as models become more complex. The training times of all the models were compared with respect to the training time per spectra. This did not include time taken to process the data.

**Figure 3:**
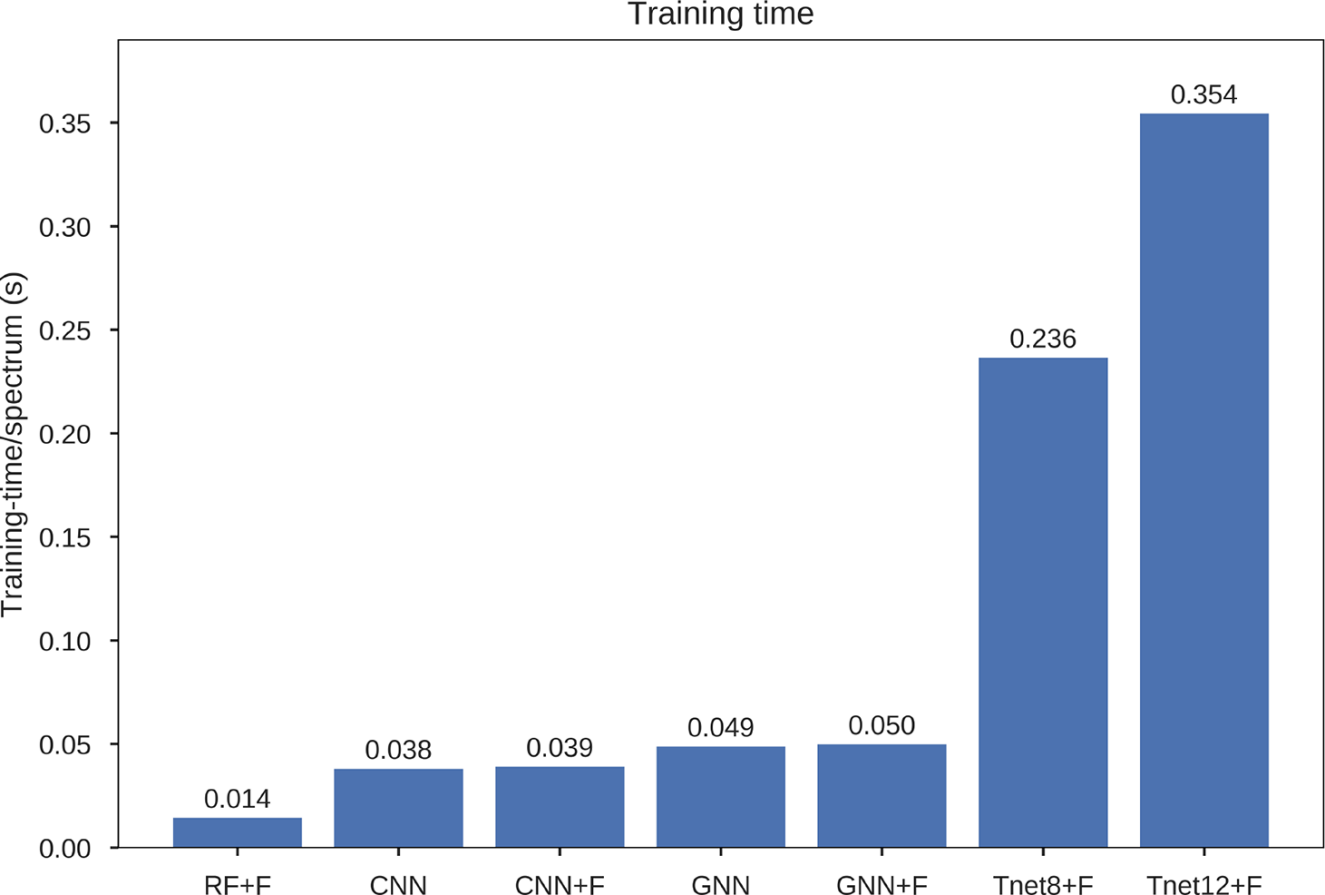
Comparison of the training times of the seven models.

Figure 3 shows the results of the time evaluation. RF was the most efficient algorithm taking 14 μs per spectrum. Both CNNs showed similar results to each other. The addition of the added features increased training time by 1 μs per spectrum from 38 μs per spectrum to 39 μs per spectrum. Both took over twice as long as the RF. The GNNs took longer to train both due to their added complexity and the difficulty in parallelising their computation. Again the addition of the extra features resulted in a 1 μs per spectrum increase in training time. Both GNNs took over 3 times longer per spectrum to train than the RF. The T Net models took substantially longer to train than any of the other models with Tnet8+F and Tnet12+F taking 236 and 354 μs per spectrum, respectively. This is due to the different way the models interpret the spectra. The other deep learning models take in candidate ion windows which are processed outside the model. Instead the T Net models use the difference from the expected ion values with some of the processing taking place inside the model itself. While this means the T Net models are slower, it shortens their data processing time making the combined time comparable to the CNNs and GNNs. The T Net data processing took approximately 2.8 μs per spectrum whereas the CNN and GNN data processing took approximately 97 μs per spectrum.

The added features caused marginal increases in training times. However, as shown in the performance evaluations earlier, they can cause large increases in prediction accuracy (Table 3). This was particularly evident for the CNN which showed a substantial improvement with the addition of the extra features. Due to their increased complexity, the GNN and GNN+F took longer to train. However, there was a substantial difference in performance (Table 3). For most accurate identification, the GNN is shown to be best suited. Nonetheless, for real-time peptide identification, the RF may be preferable due to its increased speed. When choosing the appropriate encoding module for their *de novo* algorithm, researchers must decide on the trade-off between accuracy and training time.

## Conclusion

We propose a new CNN-GNN hybrid module for peptide ion encoding. Our model was found to be more effective at identifying peptide peaks in MS spectra than the encoding modules used by the state-of-the-art algorithms, Novor, DeepNovo, and PointNovo. The CNN-GNN was better able to distinguish peptide peaks than the other modules over all levels of noise and peptide peaks present in the data. The ability of our GNN based model to incorporate long-range ion relationships yielded significantly increased performance over the other models in all data sets.

Our results suggest that there is potential for exploring the use of GNNs in *de novo* peptide sequencing algorithms. However, it is still unknown if this will improve peptide identification rates of current, state-of-the-art algorithms. To test this the encoding module described here would need to be integrated within an architecture capable of sequence prediction. One option would be combining it with an LSTM, although LSTMs require sequential vector inputs while GNNs produce an encoding for each node which do not have a natural order. Therefore, another module, such as an attention mechanism, would be required to allow the model to condense the collection of node encodings into the required sequence. Again, there are multiple ways in which this could be done, and their investigation requires further research.

The GNN module proposed in this work shows a considerable improvement in average precision (>20%) over the RF used by Novor for all data sets. These results would suggest that Novor’s methodology may become more competitive if the random forest scoring module was replaced with a more effective machine learning algorithm such as a CNN-GNN hybrid.

Finally, this research also showed the utility of Novor’s additional expert features for peptide ion identification when peaks are isolated and there are few if any connections between them. Hence our work highlights the importance of expert domain knowledge in the design of *de novo* sequencing models. Overall this research uncovers limitations in peptide ion encoding from state-of-the-art *de novo* algorithms and the presented CNN-GNN hybrid model offers a promising alternative by embedding spectral features more comprehensively.

## Supporting Information Available

Correlation between peptide spectrum features (Figure S1); effect of noise on GNN+F TPR and FPR (Figure S2); effect of noise on GNN TPR and FPR (Figure S3); effect of noise on CNN+F TPR and FPR (Figure S4); effect of noise on CNN TPR and FPR (Figure S5); effect of noise on RF+F TPR and FPR (Figure S6); effect of noise on Tnet8+F TPR and FPR (Figure S7); effect of noise on TNet12+F TPR and FPR (Figure S8); AUC for real data sets (Table S1); further discussion on AUC (PDF)
