# Supplementary material for: Application of a Novel Hybrid CNN-GNN for Peptide Ion Encoding
Source: J Proteome Res. 2022 Dec 19;22(2):323–33. doi: 10.1021/acs.jproteome.2c00234 (PMC9903319; doi:10.1021/acs.jproteome.2c00234)
Supplement: Supplementary file 1 — pr2c00234_si_001.pdf [file pr2c00234_si_001.pdf]

Supplementary Information

The application of a novel hybrid CNN-GNN for peptide ion encoding

Kevin McDonnell[a,b,*], Florence Abram[a], Enda Howley[b]

[a] *Functional Environmental Microbiology, School of Natural Sciences, Ryan Institute, University of Galway, Galway H91 TK33, Ireland*
[b] *Department of Information Technology, School of Computer Science, University of Galway, Galway H91 TK33, Ireland*
[*] *k.mcdonnell6@universityofgalway.ie*

Figure S1. Correlation of features in real tandem MS data. The correlation between the fraction of peaks present and the noise ratio in the real data used in this study is shown in A. The correlation between the length of the peptide and the noise ratio in the spectra for the same data is shown in B. Box plots indicate the distribution of spectra while the blue line indicates the mean and the green lines indicate the modes.

Figure S2. Impact of noise on the TPR and FPR of the GNN+F in artificial and real data. The average TPR is shown in green and the average FPR is shown in blue.

Figure S3. Impact of noise on the TPR and FPR of the GNN in artificial and real data. The average TPR is shown in green and the average FPR is shown in blue.

Figure S4. Impact of noise on the TPR and FPR of the CNN+F in artificial and real data. The average TPR is shown in green and the average FPR is shown in blue.

Figure S5. Impact of noise on the TPR and FPR of the CNN in artificial and real data. The average TPR is shown in green and the average FPR is shown in blue.

Figure S6. Impact of noise on the TPR and FPR of the RF+F in artificial and real data. The average TPR is shown in green and the average FPR is shown in blue.

Figure S7. Impact of noise on the TPR and FPR of the Tnet8+F in artificial and real data. The average TPR is shown in green and the average FPR is shown in blue.

Figure S8. Impact of noise on the TPR and FPR of the Tnet12+F in artificial and real data. The average TPR is shown in green and the average FPR is shown in blue.

Table S1. AUC for each model on all 9 real datasets.

Supporting Information. Further Discussion on AUC

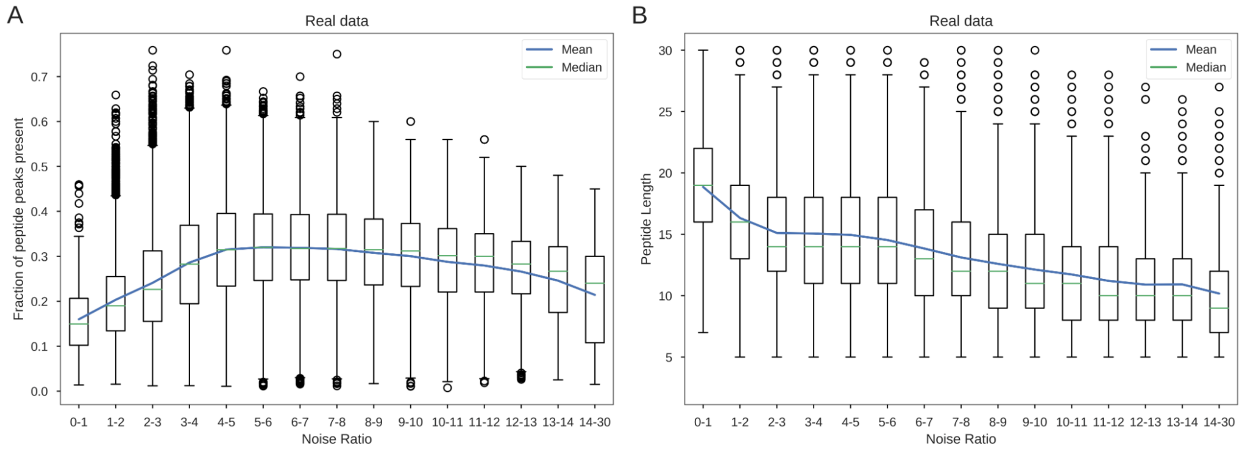

Figure S1. Correlation of features in real tandem MS data. The correlation between the fraction of peaks present and the noise ratio in the real data used in this study is shown in A. The correlation between the length of the peptide and the noise ratio in the spectra for the same data is shown in B. Box plots indicate the distribution of spectra while the blue line indicates the mean and the green lines indicate the modes.

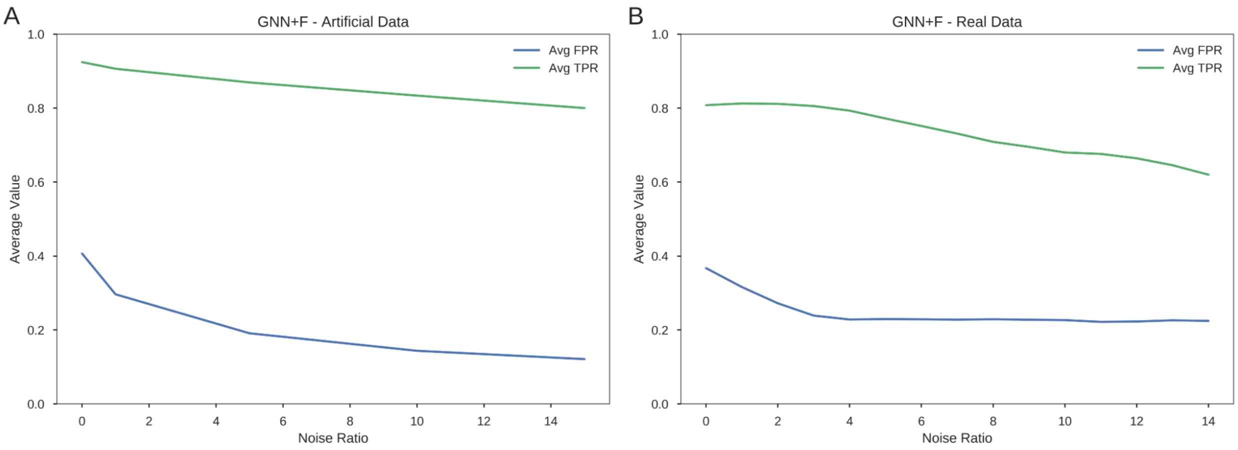

Figure S2. Impact of noise on the TPR and FPR of the GNN+F in artificial and real data. The average TPR is shown in green and the average FPR is shown in blue.

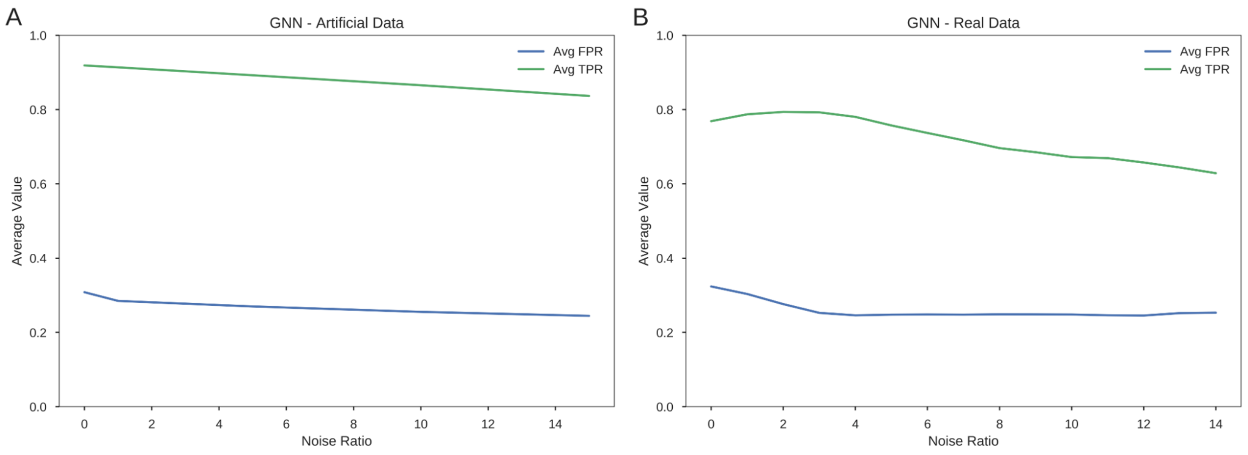

Figure S3. Impact of noise on the TPR and FPR of the GNN in artificial and real data. The average TPR is shown in green and the average FPR is shown in blue.

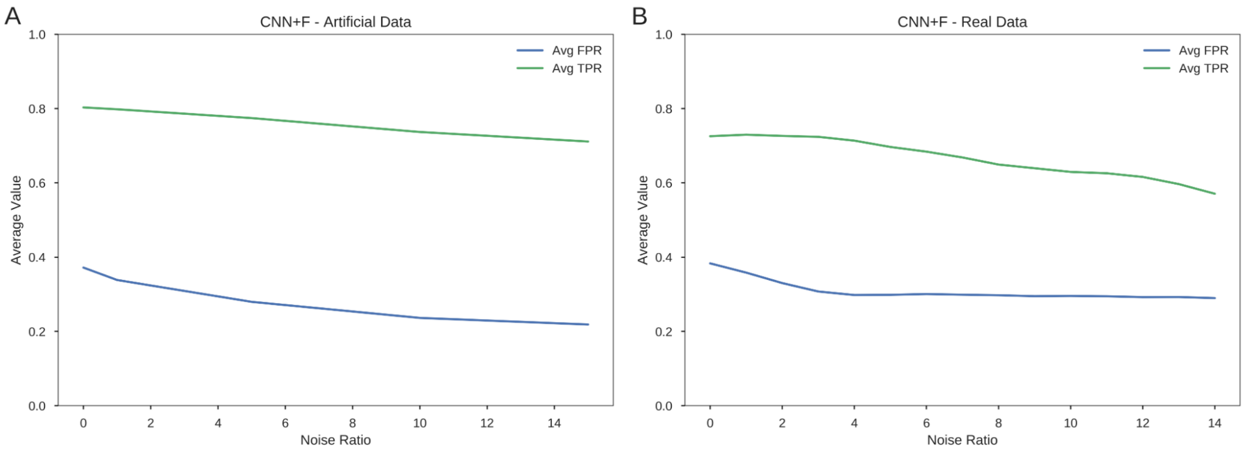

Figure S4. Impact of noise on the TPR and FPR of the CNN+F in artificial and real data. The average TPR is shown in green and the average FPR is shown in blue.

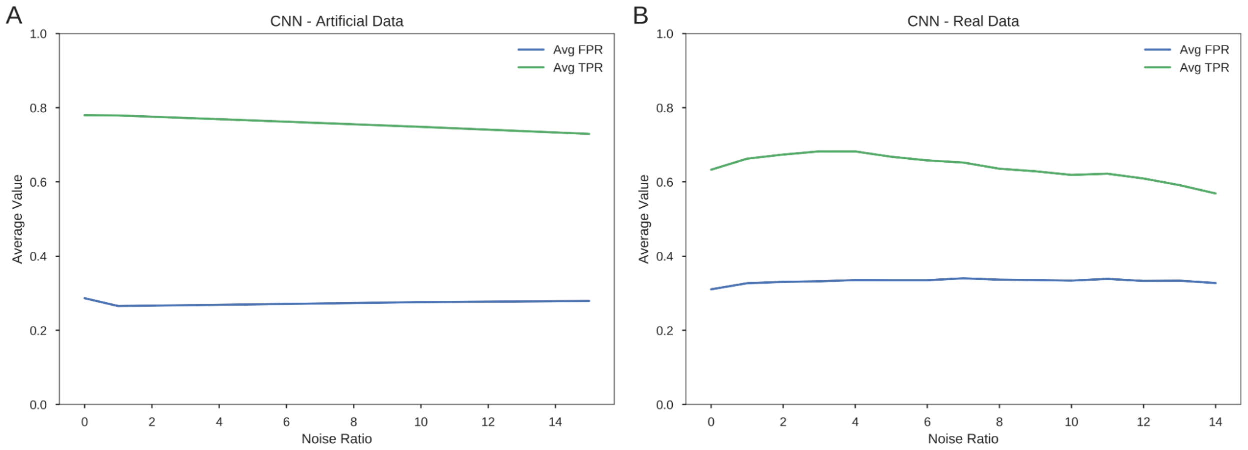

Figure S5. Impact of noise on the TPR and FPR of the CNN in artificial and real data. The average TPR is shown in green and the average FPR is shown in blue.

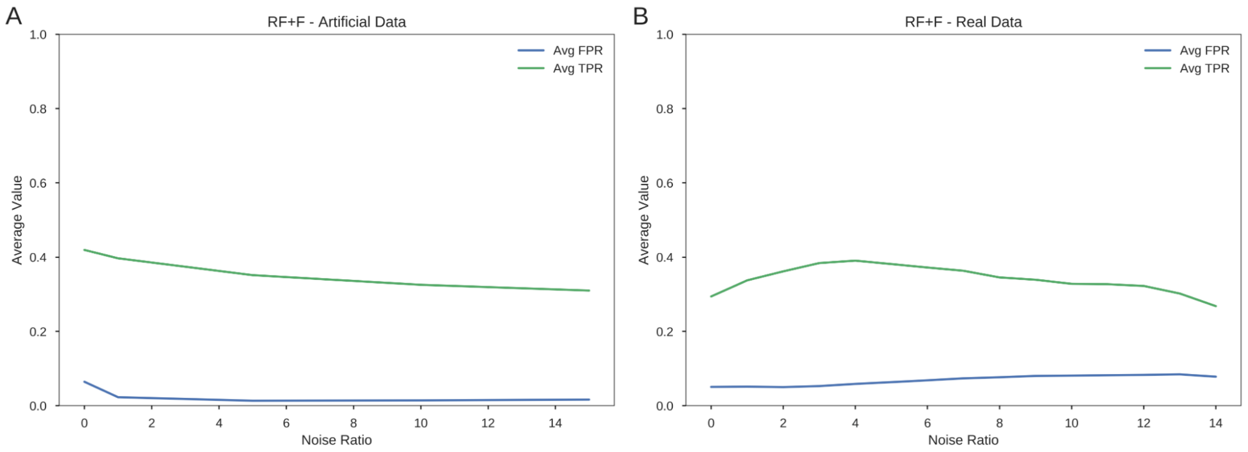

Figure S6. Impact of noise on the TPR and FPR of the RF+F in artificial and real data. The average TPR is shown in green and the average FPR is shown in blue.

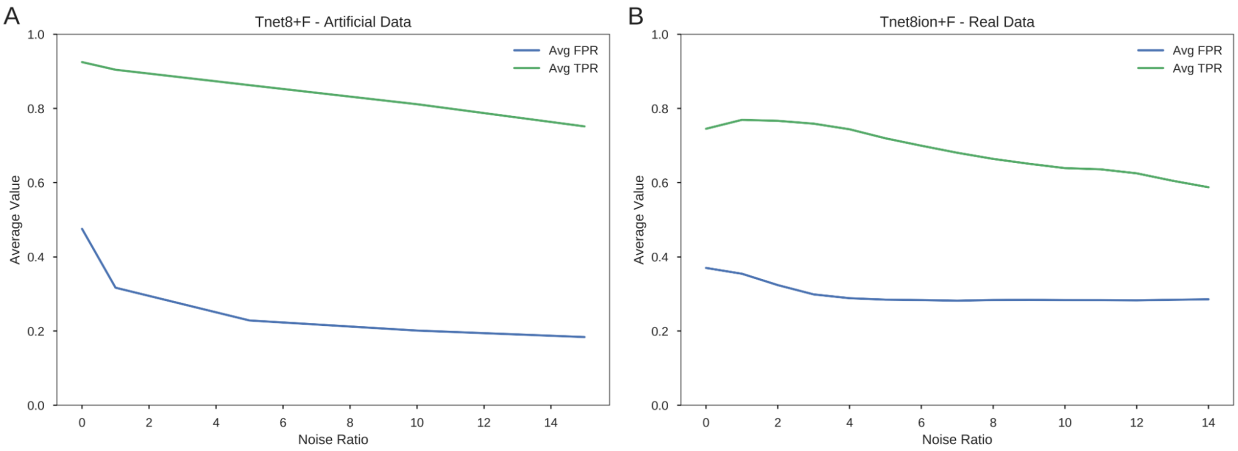

Figure S7. Impact of noise on the TPR and FPR of the Tnet8+F in artificial and real data. The average TPR is shown in green and the average FPR is shown in blue.

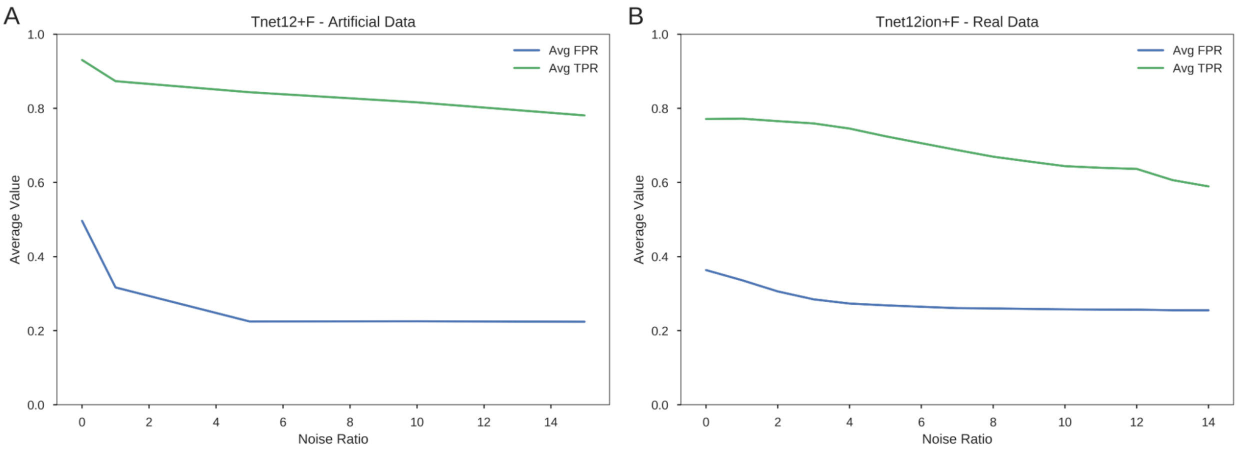

Figure S8. Impact of noise on the TPR and FPR of the Tnet12+F in artificial and real data. The average TPR is shown in green and the average FPR is shown in blue.

Dataset	**RF+F**	**CNN**	**CNN+F**	**Tnet+F 8 ion**	**Tnet+F 12 ion**	**GNN**	**GNN+F**
Yeast	0.9231	0.9354	0.9525	0.9527	0.9557	0.9702	**0.9776**
Human	0.9127	0.9193	0.9454	0.9449	0.9467	0.9585	**0.9655**
Mouse	0.8638	0.8977	0.9168	0.9221	0.9277	0.9452	**0.9511**
Bacillus	0.9078	0.9258	0.9466	0.9407	0.9454	0.9660	**0.9725**
Clam Bacteria	0.8969	0.8996	0.9337	0.9299	0.9373	0.9453	**0.9568**
Honeybee	0.8958	0.9125	0.9393	0.9329	0.9396	0.9552	**0.9654**
Ricebean	0.9092	0.9205	0.9419	0.9361	0.9431	0.9646	**0.9719**
Tomato	0.9036	0.9267	0.9464	0.9484	0.9512	0.9726	**0.9729**
M. Mazei	0.9006	0.9230	0.9436	0.9415	0.9466	0.9673	**0.9713**

Table S1. AUC for each model on all 9 real datasets.

Further Discussion on AUC

Figure S2A shows the average FPR and TPR for the GNN+F as noise was increased in the artificial data. It shows a sharp decrease in the FPR when noise is initially increased. The additional noise gives "easy" to classify examples to the models, thereby increasing AUC. Further additional noise does not keep having the same magnitude of an effect on the FPR and it levels off. The TPR drops consistently as the noise ratio increases. Additional noise makes the prediction of the positive class more difficult leading to this decrease. The decreasing TPR dominates the trends in AUC as the FPR levels off leading to the observed decrease in AUC for ratios of additional noise greater than 1. Corresponding trends were found for the other algorithms (Figures S3-8).

A somewhat similar pattern was observed when investigating real data (Figure S2B). Decreases in the FPR are present at low noise followed by a levelling off just like in the artificial data for almost every model (Figures S3-8).

Unlike the artificial data however, an increase in the TPR is observed at low noise ratios. This could be due to the aforementioned difference in the fraction of peptide peaks present for these data (Figure S1A). These low noise data are also correlated with increased peptide length which the models find more difficult to successfully classify (Figure S1B). For noise ratios above 5 when the fraction of peaks present stops increasing, TPR decreases just as it did in the artificial data for increasing noise.

While these AUC results may not have been expected, the rank order of the models remained fairly consistent with that of average precision (Table S1). This suggests AUC did a reasonable job at sorting the models based on competence. However, the analysis shows how care should be taken when comparing AUC across datasets, particularly if the class distribution is different between datasets.
